# The Gαq/11 Proteins Contribute to T Lymphocyte Migration by Promoting Turnover of Integrin LFA-1 through Recycling

**DOI:** 10.1371/journal.pone.0038517

**Published:** 2012-06-11

**Authors:** Lena Svensson, Paula Stanley, Frances Willenbrock, Nancy Hogg

**Affiliations:** Leukocyte Adhesion Laboratory, Cancer Research UK London Research Institute, London, United Kingdom; University of Birmingham, United Kingdom

## Abstract

The role of Gαi proteins coupled to chemokine receptors in directed migration of immune cells is well understood. In this study we show that the separate class of Gαq/11 proteins is required for the underlying ability of T cells to migrate both randomly and in a directed chemokine-dependent manner. Interfering with Gαq or Gα11 using dominant negative cDNA constructs or siRNA for Gαq causes accumulation of LFA-1 adhesions and stalled migration. Gαq/11 has an impact on LFA-1 expression at plasma membrane level and also on its internalization. Additionally Gαq co-localizes with LFA-1- and EEA1-expressing intracellular vesicles and partially with Rap1- but not Rab11-expressing vesicles. However the influence of Gαq is not confined to the vesicles that express it, as its reduction alters intracellular trafficking of other vesicles involved in recycling. In summary vesicle-associated Gαq/11 is required for the turnover of LFA-1 adhesion that is necessary for migration. These G proteins participate directly in the initial phase of recycling and this has an impact on later stages of the endo-exocytic pathway.

## Introduction

Small chemoattractant peptides called chemokines direct T lymphocytes (T cells) to arrest on post-capillary venules at sites of infection or injury [1], [2]. Chemokines bind to G protein-coupled receptors (GPCRs), initiating signalling that activates integrins such as lymphocyte function-associated antigen-1 (LFA-1, CD11a/CD18, αLβ2) [3], [4]. The chemokine GPCRs are coupled to heterotrimeric G proteins composed of α, β and γ subunits and signal through active Gαi-GTP and Gβγ dimers leading to generation of intracellular effectors such as Ca^2+^ and diacylglycerol [5], [6]. One of the key downstream effectors of chemokine triggered signalling is the GTPase Rap1. It has several critical roles in LFA-1 activation that lead to arrest of circulating T cells onto vessels and their subsequent firm adhesion to and migration along the vessel walls and into tissue [4], [7].

Other groups of G proteins such as the Gαq/11 family comprising Gαq, 11, 14 and 15/16, have also been implicated in immune cell functions such as migration but less is known about how they mediate their effects compared with Gαi proteins [6], [8]. Gαq and Gα11 are widely expressed and are the most homologous members of this family with many of their activities considered to be over-lapping. There are conflicting reports about the involvement of these Gαq/11 proteins in migration. A positive role was demonstrated by the failure both of Gα11-inhibited myeloid leukaemia cells to migrate to lymphoid tissues and of the LFA-1-mediated tissue invasion of a Gα11-inhibited T cell hybridoma [9], [10]. Similarly neutrophils and dendritic cells from mice lacking Gαq (*Gnaq* ^−/−^) have deficient chemotactic responses, with the defect apparently not extending to *Gnaq* ^−/−^ T cells [11]. In contrast Gαq siRNA-mediated knockdown in the Jurkat T cell line enhanced migration in response to chemokine CXCL12 suggesting a repressive effect of the Gαq protein on motility [12].

In this study we have focussed on the role of Gαq and Gα11 in T cell migration mediated by the integrin LFA-1. Blocking Gαq/11 activity increased LFA-1-mediated adhesion and led to a reduction in the ability of T cells to migrate both randomly and towards chemokine. We show that this G protein family is required for the turnover of LFA-1 adhesions, has a specific role in their endocytosis and has an impact beyond its expression in the intracellular trafficking of LFA-1.

## Results

### Gαi2 is Needed for Directed but not Random Migration

**Figure 1:**
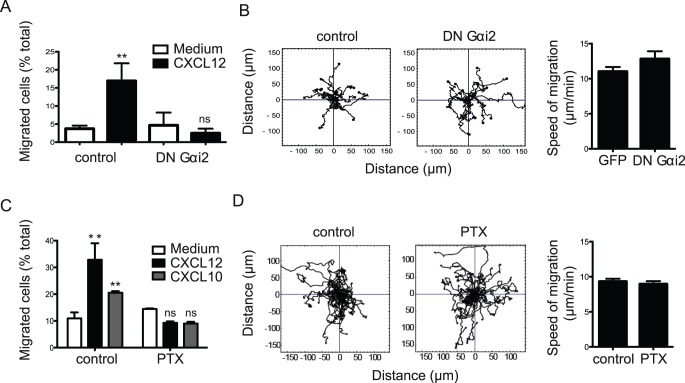
Gαi2 is needed for directed but not random migration. (**A**) HSB2 T cells ± transfection with DN Gαi2 or vector control cDNAs showing chemotactic response to CXCL12 (SDF-1α) or medium alone using a Transwell assay, n = 4 experiments; (**B**) Random migration of HSB2 T cells on ICAM-1 treated as (A): a representative experiment of single cell tracking patterns (n = 15 cells per condition) and collective speed of migration (mean ± s.d.), n = 3 experiments; (**C**) T lymphoblasts ± pre-treatment with pertussis toxin (PTX) at 200 ng/ml showing chemotactic response to CXCL12, CXCL10 (IP-10) or medium alone using a Transwell assay, n = 3 experiments; (**D**) Random migration on ICAM-1 of T blasts treated as (C): a representative experiment of single cell tracking patterns (n = 25 cells per condition) and collective speed of migration (mean ± s.d.), n = 3 experiments.

To investigate involvement of different classes of heterotrimeric G proteins in T cell migration, we first asked whether the HSB2 T cell line was able to respond to a chemoattractant by testing its migration toward CXCL12 (SDF-1α) in a Transwell assay. Transfection of T cells with a dominant negative (DN) cDNA construct of the G protein, Gαi2, which is involved in chemokine-mediated chemoattraction [13], caused decreased migration towards CXCL12 (87±4% decrease) (**Fig. 1A**). Placing CXCL12 in both upper and lower wells abrogated the directed movement of the T cells indicating that a chemotactic effect was being detected (data not shown). The transfection did not affect membrane expression of CXCR4, the CXCL12 receptor, compared with T cells transfected with vector control (data not shown).

Although unable to respond to the chemokine, T cells transfected with DN Gαi2 cDNA had however the same capacity as cells expressing the vector control to migrate randomly on surfaces coated with the LFA-1 ligand, intercellular adhesion molecule-1 (ICAM-1) (**Fig. 1B**). There was no difference in either directionality or speed of migration between DN Gαi2-treated versus vector control-treated T cells (DN Gαi2, 12.8±1.1 µm/min; control, 11.0±0.7 µm/min (mean±s.d.)).

To ask whether this distinction between directed and random migration applied more generally and to rule out autocrine chemokine stimulation, we treated T lymphoblasts with pertussis toxin (PTX) that inhibits Gαi activity by catalyzing the ADP-ribosylation of Gαi proteins. PTX had significant impact on directed migration towards chemokines CXCL12 and CXCL10 (IP-10) as expected (% decrease: CXCL12, 71.5±3.5%; CXCL10, 56.5±1.5%) (**Fig. 1C**). However, as with HSB2 T cells expressing DN Gαi proteins, the ability of T lymphoblasts to migrate randomly on ICAM-1 with regard to either directionality or the speed of migration was unaffected by PTX (PTX, 9.0±0.6 µm/min; control, 9.3±0.6 µm/min (mean±s.d.)) (**Fig. 1D**). These findings suggest that the Gαi-containing heterotrimers are not utilized for random migration of T cells.

### The Gαq/11 Subgroup is Needed for both Random and Directed T Cell Migration

**Figure 2:**
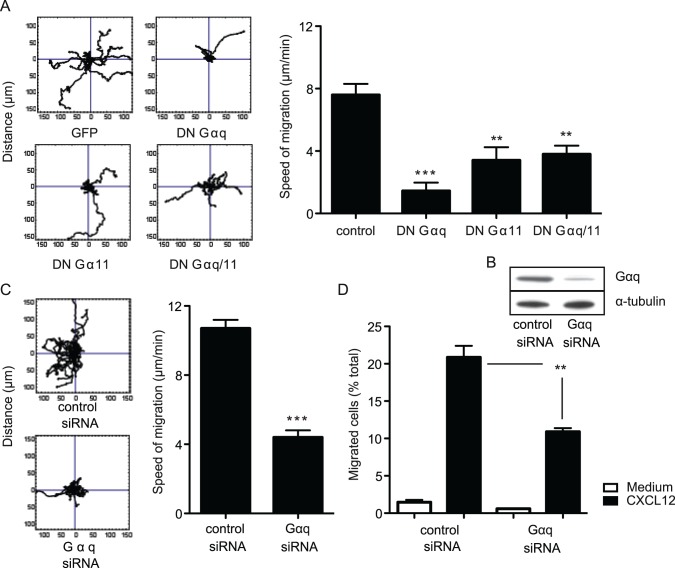
The Gαq/11 proteins control both random and directed T cell migration. (**A**) Random migration on ICAM-1 of HSB2 T cells ± transfection with vector control or DN Gαq, DN Gα11 or both cDNAs showing a representative experiment of single cell tracking patterns (n = 15 cells per condition) and speed of migration (mean ±s.d.), n = 5 experiments; (**B**) Western blot showing expression level of Gαq ± treatment of HSB2 cells with either control siRNA or Gαq siRNA and α-tubulin to represent the sample loading control; (**C**) Primary T cells treated with either control siRNA or Gαq siRNA showing single cell tracking pattern (n = 15 cells per condition) and random migration on ICAM-1 (mean ± s.d.), n = 3 experiments; (**D**) Transwell assay showing extent of chemotactic response to CXCL12 of primary T cells treated with either control siRNA or Gαq siRNA, n = 3 experiments.

We next investigated the effect on migration of the Gαq/11 subgroup of heterotrimeric G proteins whose function is insensitive to PTX [6]. DN cDNA constructs for the two major members of this group, Gαq and Gα11, had significant impact on LFA-1-dependent migration when transfected into HSB2 T cells either singly or together. Both the extent of single cell tracking and overall speed were substantially reduced (DN Gαq, 1.5±0.5 µm/min; DN Gα11, 3.4±0.9 µm/min; DN Gαq/11, 3.8±0.6 µm/min; control, 7.9±0.7 µm/min) (mean±s.d.)(**Fig. 2A**).

To further confirm the role of these G proteins by a means alternative to cDNA transfection, we focussed on Gαq that gave the most robust siRNA knockdown. For Gαq siRNA-treated HSB2 T cells, Gαq protein was reduced by ∼85% to a level of 16.5±1.8% of control siRNA-treated cells (mean±s.d. of n = 5 experiments) (**Fig. 2B**). For primary T cells the average level of Gαq siRNA knockdown was ∼60% with Gαq expression reduced to 40.0±8.9% of control siRNA (mean±s.d. of n = 3 experiments). A significant decrease in single cell tracking and speed of random migration was observed with siRNA-treated primary T cells (Gαq siRNA, 4.4±0.4 µm/min, control siRNA, 10.7±0.5 µm/min) (**Fig. 2C**).

We next examined a possible role for Gαq in migration directed by chemokines. Gαq siRNA-treated primary T cells displayed a decrease of 41±6% in chemotaxis to CXCL12 in a Transwell assay compared with control siRNA-treated T cells (**Fig. 2D**). An equivalent result was obtained when HSB2 T cells were transfected with DN Gαq/11 and tested for chemotaxis to CXCL12. The transfected cells were reduced to the background levels of migration (**Supplementary Fig. S1**).

It was important to evaluate any role for Gαq/11 proteins in a shear flow assay that tests the ability of integrins to attach under conditions of mechanical stress as experienced in the circulation. The rolling and attaching behavior of Gαq siRNA-treated HSB2 T cells was assessed on chambers coated with ICAM-1 and E-selectin at a shear force of 1 dyne as previously described [14]. Both Gαq and control siRNA-treated T cells rolled normally on E-selectin and were able to attach both transiently and firmly to ICAM-1 (**Supplementary Fig. S2A**). The ability of Gαq siRNA-treated cells to adhere normally was further confirmed in a static adhesion assay where DN Gαq/11-transfected T cells adhered to ICAM-1 comparably to the control T cells (**Supplementary Fig. S2B**).

Thus the Gαq/11 proteins have a role in the random migration of T cells and also when the cells are undergoing directed migration to a chemoattractant such as a chemokine. However the G proteins appear not to influence T cell adhesion to ICAM-1 under either static or mechanical shear conditions.

### DN Gαq/11 Reduction Alters the Morphology of ICAM-1-adhered T Cells

**Figure 3:**
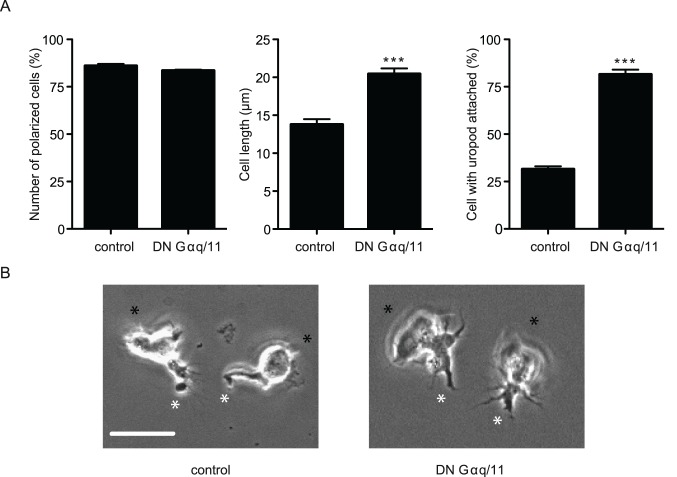
DN Gαq/11 alters the morphology of ICAM-1-adhered T cells. (**A**) HSB2 T cell transfected with DN Gαq/11 or vector control cDNAs showing morphology following attachment to ICAM-1 for 20 min at 37°C. Effect on polarization, cell length and cell uropod attachment to ICAM-1 (n = 25 cells per sample); results expressed as mean ± s.d.; (**B**) Representative live cell images of ICAM-1 attached T cells following transfection with vector control cDNA (left) showing elevated uropods or DN Gαq/11 cDNAs (right) with attached trailing edge and uropods; black asterisk = leading edge, white asterisk = trailing edge; scale bar = 10 µm.

To gain further understanding of how signaling through Gαq/11 might regulate LFA-1-mediated migration, we investigated the morphology of T cells transfected with either a combination of DN Gαq/11 cDNAs or vector alone. The DN Gαq/11-treated T cells were polarized to the same extent as control T cells (DN Gαq/11, 83.5±0.7% versus control, 86±1.5%) (**Fig. 3A**). However, DN Gαq/11-transfected T cells displayed increased total cell length compared with control T cells (DN Gαq/11, 20.5±3.5 µm versus control, 13.85±3.5 µm (mean±s.d.)). Additionally the uropods of the majority of DN Gαq/11-transfected T cells were attached to the ICAM-1-coated surface rather than elevated above it (DN Gαq/11, 81.5±2.5% versus control, 31.5±1.5%). Live cell images highlighted an abnormally attached rear of the DN Gαq/11-transfected T cells providing further evidence that T cells lacked the ability to detach correctly (**Fig. 3B** **, Supplementary Videos S1 and S2**). A lack of effect on migration of T cells transfected with WT Gαq cDNA provided further evidence that the DN cDNAs were working as expected (data not shown).

These observations indicate that T cells transfected with DN Gαq/11 cDNAs are able to move the leading edge of the cell forward but have impaired ability to detach from the substrate ICAM-1.

### Association of Gαq/11 with LFA-1 Endocytosis and Intracellular Vesicles

**Figure 4:**
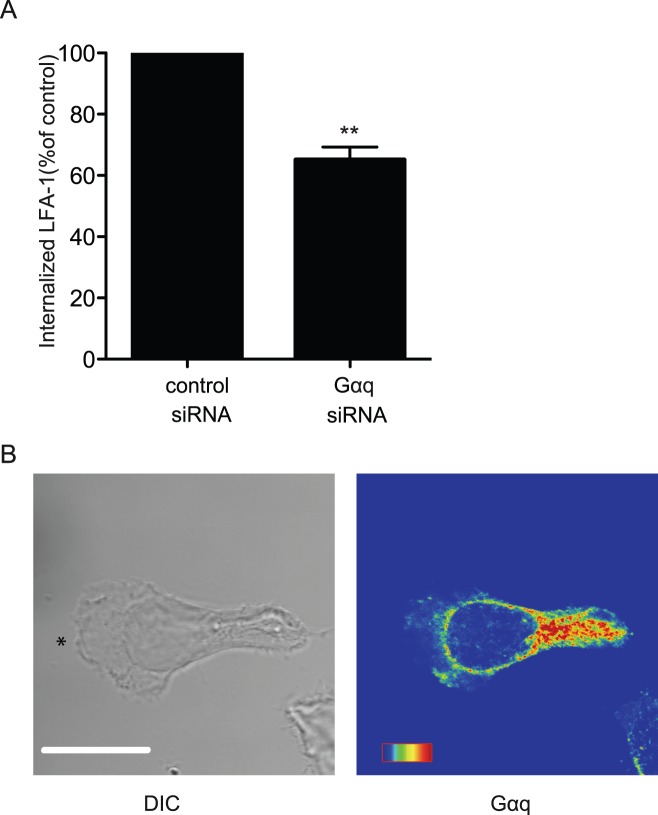
Association of Gαq/11 with intracellular vesicles and LFA-1 internalization. (**A**) Biotinylated HSB2 T cells migrating on ICAM-1 over 40 min with quantification of the effect on membrane LFA-1 internalization of transfection with siRNA Gαq compared with control siRNA, n = 3 experiments; (**B**) Representative confocal microscopy image at substrate level of T lymphoblast polarized on ICAM-1 and fixed before staining with rabbit anti-Gαq and Alexa488 goat anti-rabbit IgG; shown with Rainbow scale of graded fluorescence intensity; black asterisk = leading edge; scale bar = 10 µm;

An association has been made previously between the failure of LFA-1 detachment and β2 subunit mutation leading to lack of LFA-1 endocytosis [15]. It was therefore of interest to test whether there was a connection between the ability of the Gαq/11-inhibited T cells to turnover LFA-1 adhesions and endocytosis or recycling of this integrin. To investigate LFA-1 internalization biochemically, intact HSB2 T cells were surface labelled with glutathione-cleavable biotin and allowed to migrate on ICAM-1 for 40 min followed by LFA-1 immunoprecipitation and blotting for biotinylated integrin. By removal of membrane LFA-1 using glutathione, total LFA-1 could be distinguished from internalized LFA-1 [16], [17]. The T cells were also treated with primaquine (PQ), which is a lysosomotrophic amine that slows recycling by blocking membrane fusion of exocytic vesicles [17]. When Gαq siRNA-treated T cells were compared with control siRNA-treated cells there was a 35±4% reduction in internalized LFA-1 based on total cell biotinylated LFA-1 levels (**Fig. 4A**).

Confocal microscopic images of T blasts revealed a pattern of Gαq-expressing intracellular vesicular structures with highest density of immunostaining in the juxta-nuclear region of the polarized cells and trailing edge with scattered distribution towards the front of the cell (**Fig. 4B**). Thus Gαq not only influences LFA-1 internalization, but is also associated with intracellular vesicles. These observations provide the first suggestion that the Gαq proteins might be involved in LFA-1 recycling.

### Co-localization of Gαq with LFA-1 and Vesicle Markers

**Figure 5:**
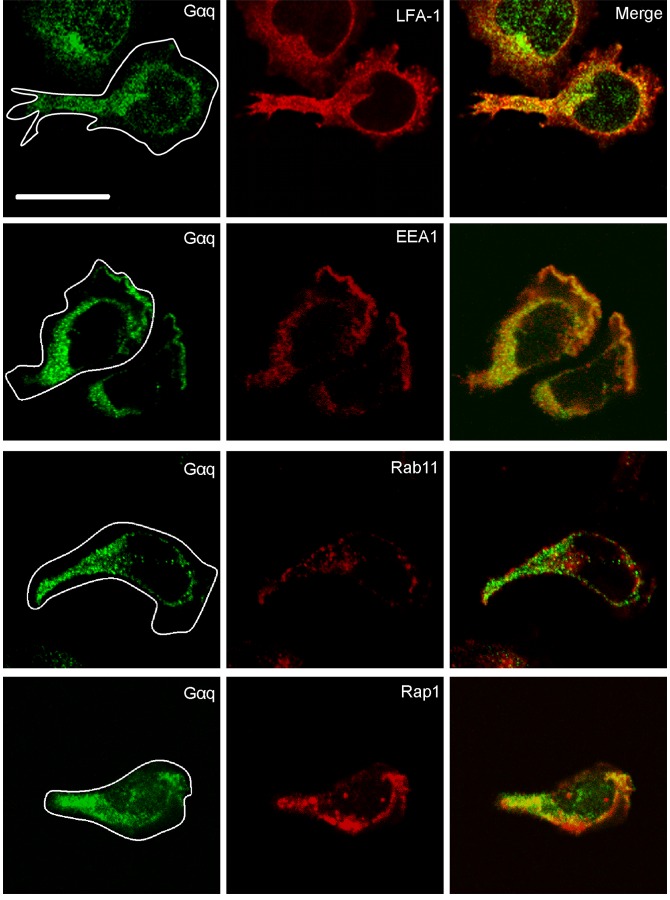
Co-localization of Gαq with LFA-1 and vesicle markers. Confocal images of T lymphoblasts attached to ICAM-1. A comparison is made of Gαq localization (green) with LFA-1 and intracellular markers of endosomal vesicles EEA-1, Rab11 and Rap1 (all red) and subsequent merged images; the images are representative of n = 4 experiments; scale bar = 10 µm.

We used confocal microscopy to further define the association of Gαq with LFA-1 and other intracellular vesicle markers. Gαq co-localized with LFA-1 particularly prominently where endosomal vesicles are located in the juxta-nuclear region and trailing edge (**Fig. 5**). Pixel-by-pixel analysis yielded 95.4±1.8% overlap between Gαq and LFA-1 (n = 5 cells). Gαq staining also overlapped with EEA1, a key Rab5 effector protein [18] (Overlap analysis = 94.2±4.0%, n = 5 cells). However, although it was well-expressed, there was essentially no overlap of Gαq with Rab11, a marker of a subset of late endosomal vesicles (Overlap analysis = 12.7±3.9%, n = 5 cells) and previously associated with LFA-1 recycling [16].

The GTPase Rap1 is required for the early events of LFA-1 adhesion [19] and also with its transport in intracellular vesicles [4], [7], [20], [21]. We observed a partial overlap of Gαq with Rap1 (Overlap analysis = 67.7±12.5%, n = 5 cells) suggesting that Rap1 has associations beyond those with Gαq.

In summary, confocal microscopy showed that there was selectivity in immunostaining of Gαq in that it overlapped with LFA-1 and with some, but not all, endosomal vesicle markers previously associated with this integrin.

### Association of Gαq with LFA-1 and Vesicle Markers at Plasma Membrane Level

**Figure 6:**
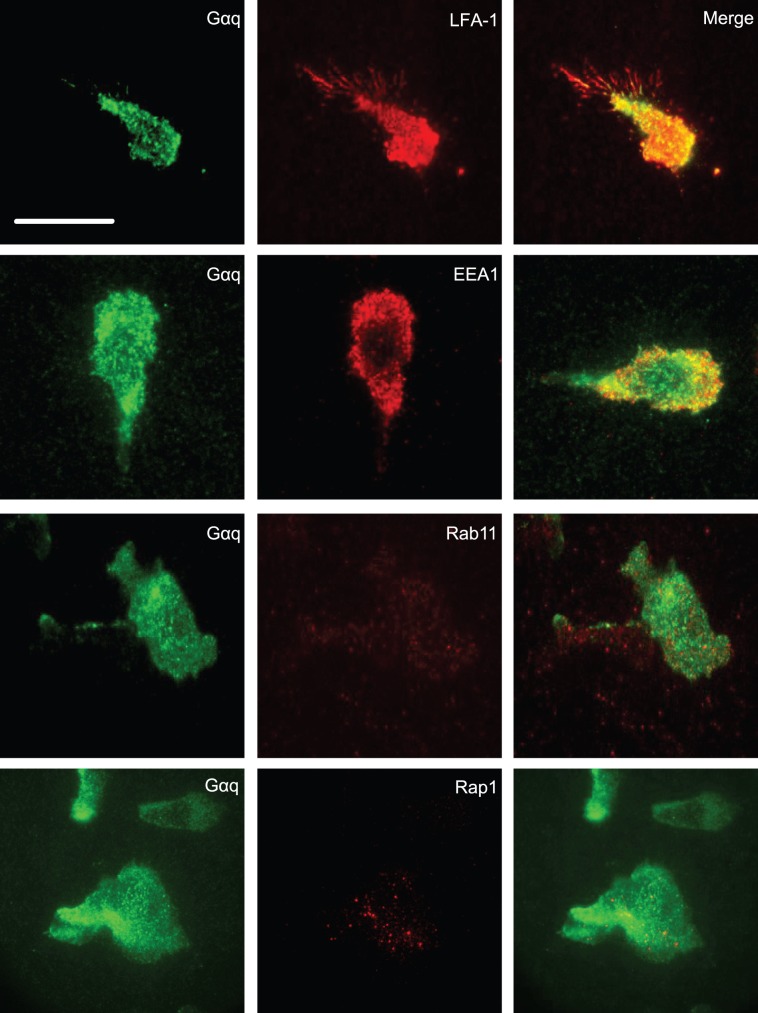
TIRF images of T lymphoblasts polarized on ICAM-1. Single cell images of T lymphoblasts viewed at ICAM-1 level by TIRF showing co-localization of Gαq expression (green) with LFA-1 and endosomal vesicle markers EEA1 (red) and merge of both colors. There is an absence of detection of Rab11 and Rap1 at TIRF level; the presented images are from the same experiment as illustrated in Fig. 5; images are representative of n = 3 experiments; scale bar = 10 µm.

We next used TIRF microscopy to look in closer detail at the T cell membrane where LFA-1 was in contact with ICAM-1. At TIRF level, Gαq co-localized with LFA-1 in the main cell body where the integrin attached to ICAM-1 sparing the attached filopodia (Overlap analysis = 93.8±6.2%, n = 5 cells). Co-localization of Gαq staining with EEA1 was also observed (Overlap analysis = 81.6±12.9%, n = 5 cells) (**Fig. 6**). Thus Gαq was expressed at membrane level with LFA-1 and EEA1. In contrast, Rab11 and Rap1 were both poorly visible at TIRF level. As both proteins were easily imaged at epifluorescence level, the implication is that they are expressed intracellularly but away from the plasma membrane.

### Effect of Gαq Reduction on Vesicle Expression and Distribution

**Figure 7:**
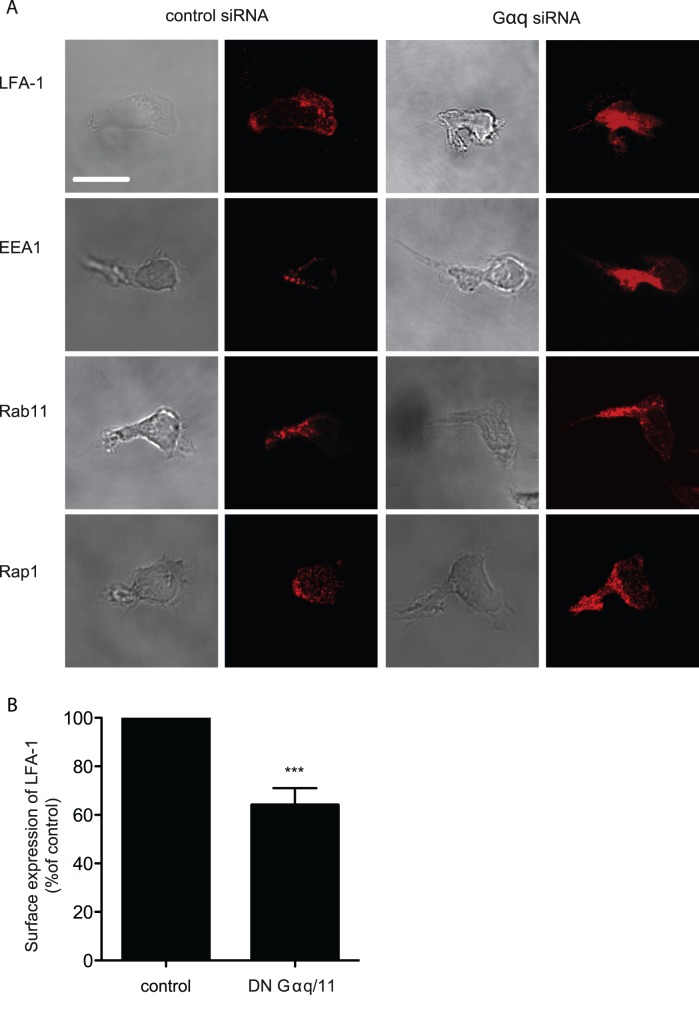
Effect of Gαq siRNA knockdown on intracellular vesicles. (**A**) DIC and confocal microscopy images of HSB2 T cells on ICAM-1 showing distribution and intensity of staining of LFA-1, EEA1, Rab11 and Rap1 following transfection with either control or Gαq siRNAs; results are representative of n = 3 experiments, scale bar = 10 µm; (**B**) Comparison of membrane LFA-1 expression per comparable cell area in T cells treated either with control or DN Gαq/11 cDNAs.

It was relevant to ask whether Gαq siRNA knockdown altered the expression or distribution pattern of intracellular vesicles. When Gαq siRNA-treated T cells were compared with control siRNA-treated cells, it was evident that the T cells with reduced Gαq displayed increased expression of LFA-1 and EEA1 that were both co-expressed with Gαq (**Fig. 7A** **,** **Table 1**). In addition, although Gαq only partially overlapped with Rap1, and not at all with Rab11, the staining associated with these vesicle markers was also increased in the Gαq siRNA-treated T cells. Therefore the reduction in Gαq expression had an impact not only on Gαq -expressing vesicles, but also on vesicles with which it did not co-distribute.

**Table 1:** Quantification of the effect of Gαq siRNA knockdown on expression of other vesicle markers.

	control siRNA	Gαq siRNA	P value
LFA-1	705±5*	739±5	0.0014
EEA1	679±7	710±4	0.0018
Rab11	696±3	737±10	0.0034
Rap1	662±16	719±8	0.015

Mean fluorescence intensity (MFI) measurements were quantified from 10 T cells per sample type for integrin LFA-1, Rab5 effector EEA1 and GTPases Rab11 and Rap1. The HSB2 T cells were transfected with either control or Gαq siRNAs for 48 hr prior to attaching to and migrating on ICAM-1.

* Mean fluorescence intensity ± SD.

Finally it might be expected that these effects of lack of Gαq on vesicular traffic would have an impact on membrane expression of LFA-1. Using laser scanning cytometry to examine LFA-1 expression per unit cell area, a comparison of T cells transfected with control versus DN Gαq/11 cDNAs revealed ∼40% reduction in expression of LFA-1 on membranes of Gαq/11 compromised T cells consistent with intracellular accumulation of recycling vesicles (**Fig. 7B**).

The accumulation of several types of intracellular vesicles when the level of Gαq is diminished is consistent with there being a general slowdown in intracellular trafficking of LFA-1 that has a knock-on effect of causing reduction in LFA-1 at membrane level.

## Discussion

In this study we find that the Gαq/11 class of G proteins has an essential function in the LFA-1-mediated migration of human T cells. These two G proteins are needed for a basic aspect of migration as their blockade affects both random migration and also when the T cells are undergoing directed chemokine-mediated migration through the use of G protein, Gαi2. The use of dominant negative cDNAs indicate an overlapping role for Gαq and Gα11 as has been previously suggested [6], [8]. We provide evidence that Gαq/11 proteins participate in LFA-1 recycling and in particular that they regulate LFA-1 adhesion turnover, internalization and membrane expression. The effect on migration is in keeping with an earlier report indicating Gα11 involvement in the LFA-1-mediated migration and invasion of a T cell lymphoma [10]. Furthermore there is increasing evidence that recycling of integrin is generally essential for successful migration of leukocytes. For example, the presence of α5β1 [22], [23] and LFA-1 [16] in recycling vesicles drives neutrophil migration.

An initial observation was that blockade of Gαq and Gα11 activity or Gαq siRNA knockdown caused excessive T cell adhesion to ICAM-1. Although the leading edge of the T cells displayed some forward motility, migration halted because of increased attaching, not at the front, but at the rear of the cell. Blocking Gαq activity however had no overall effect on LFA-1-mediated adhesion of T cells under either static or shear flow conditions, suggesting that the pro-adhesive phase was not affected. The evidence thus pointed to an inability of Gαq/11-compromised T cells to detach or turnover their LFA-1 adhesions.

A previous report showed that mutation of the β2 subunit in the endocytosis motif prevented not only endocytosis, but also LFA-1 turnover implying a connection between the two processes [15]. Similarly we found that Gαq affected de-adhesion and was also required for LFA-1 internalization. This was consistent with confocal images showing the presence of Gαq on intracellular vesicles and co-localization with LFA-1 and EEA1, a protein associated with the GTPase Rab5 that characterizes vesicles involved in the early endocytic stage of recycling [24].

The GTPase Rap1 plays an essential early role in lymphocyte arrest on the vasculature following chemokine stimulation [19] and this is consistent with its involvement in vesicle transport as well-demonstrated in other studies [20], [21], [25], [26]. Gαq only partially co-distributed with Rap1 suggesting additional roles for the GTPase. Other studies report Rap1 to be associated with EEA1, Rab5 and Rab11 [21] or alternatively on vesicles with limited EEA1 and Rab7 overlap [26]. Thus, in terms of vesicle marker expression, the pattern of Rap1 expression is distinct from but overlapping with Gαq. It is of interest that Rap1 is also required for delivery of LFA-1 to the membrane [27] whereas we found no role for Gαq in this activity.

Gαq did not co-distribute with Rab11 that is displayed by a subset of late endosomal vesicles. This is of relevance because LFA-1 recycling has also been associated not only with Rap1, but with recycling vesicles expressing Rab11 and with trafficking of LFA-1 to the lamellipodia [16]. The data therefore support the idea that Gαq is associated with the initial endocytic phase of LFA-1 recycling and not with the later stages. Thus there must be heterogeneity in LFA-1-transporting vesicles with Gαq characterizing one set and Rap1 and Rab11, a partially overlapping and separate set respectively that are involved in the subsequent events of bringing LFA-1 to the membrane followed by exocytosis. Supporting evidence comes from a comparison between epifluorescence and TIRF observations that show both Rap1 and Rab11 to be most highly concentrated away from the level of close membrane contact of T cell LFA-1 with ICAM-1. The Rab11-dependent release of LFA-1 into membrane ruffles at the leading edge of CHO cells is in keeping with their lack of substrate contact [16].

The influence of Gαq on the behavior of intracellular vesicles however goes beyond the vesicles that express it, as Gαq siRNA knockdown affects not only EEA1-expressing vesicles on which Gαq is co-expressed, but also Rap1- and Rab11-expressing vesicles. These vesicle markers normally display a juxta-nuclear distribution pattern with scattered representation towards the front of the polarized T cell. In Gαq siRNA-treated T cells, the juxta-nuclear pattern is relaxed and this is accompanied by an increase in vesicles expressing not only LFA-1 and EEA1, but also Rap1 and Rab11. Such a “log jam” of vesicles and the reduction in LFA-1 internalization all point to a disturbance in the LFA-1 recycling network that follows on from reduction in Gαq with consequences beyond the vesicles that normally express it. Thus the findings indicate that the Gαq-expressing vesicles are linked in terms of function to other vesicles with later involvement in the endocytic/exocytic sequence of events involved in the intracellular movement of LFA-1 back to the membrane. An end result of the failure of normal recycling is the ∼40% decrease in expression of membrane LFA-1. This is apparently not fully compensated for by a decrease in LFA-1 internalization in Gαq/11 blocked cells. It was also not reflected in diminished adhesion, but rather the reverse, consistent with disturbance in LFA-1 turnover as evident from impaired uropod retraction.

Some of these activities of Gαq/11 are similar to those of the Gα12/13 class of G proteins. T cells from Gα12/13-deficient mice also display increased LFA-1-mediated adhesion [28]. A speculation is that Gα12/13 may also be involved in recycling of integrin as Rab11-expressing vesicles that are Gα13-dependent are associated with an intracellular recycling compartment of T cells containing CXCR4/TCR heterodimers [29].

In summary we here define an early stage in LFA-1 recycling on T cells that is regulated by the Gαq/11 proteins. Although Gαq/11 has a direct role in endocytosis, there is evidence for more extended influence on an interconnected sequence of events involving other types of vesicles that traffic LFA-1 back to the T cell membrane. The presented evidence does not exclude the existence of LFA-1 recycling pathways that are completely independent of Gαq/11. A key issue for the future will be to determine the relationship between the different LFA-1-containing intracellular vesicles and the extent of their heterogeneity.

## Materials and Methods

### Antibodies and Reagents

MAbs used in this study are: 38, pan-LFA-1, prepared at CRUK LRI [30]; 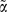 tubulin DM1A (T6199, Sigma-Aldrich Ltd, Gillingham, Kent, UK); GTPase Rap1 (610196), endosomal vesicle markers EEA1 (610457) and Rab11 (610656) (all BD Transduction Laboratories, Oxford UK); rabbit anti- Gαq Ab (E-17) (SC-393, Santa Cruz Biotechnology Inc/Insight Biotechnology Ltd, Wembley, UK). Five domain ICAM-1Fc was produced as previously described [31].

### Cell Isolation, Culture and Transfection

Peripheral blood mononuclear cells were prepared from single donor leukocyte buffy coats (National Blood Service, Tooting, London, UK); T cells were expanded as previously described and used between days 10 and 14 [31]. Primary T cells were isolated using the MACS Pan T cell isolation kit II by negative depletion (Miltenyi Biotec GmbH, Bergisch Gladbach, Germany). The human T lymphoblast CD3^−^ T cell line, HSB2, isolated from an acute lymphoblastic leukemia source (ATCC number CCL-120.1, known as CCRF-HSB-2 or HSB2) was maintained in RPMI 1640/10% FCS [32].

HSB2 T cells (2×10^7^ cells) were washed in OptiMEM + GlutaMAX (Invitrogen, Paisley, UK) and electroporated with the following reagents all at 400 nM per reaction using a Gene Pulser with Capacitance Extender (Bio-Rad UK, Hemel Hempstead, UK) set at 960 µF and 300 mV: Gαq siRNAs (Experimentally Verified GNAQ5 and GNAQ6) or negative control siRNA (all Quiagen Ltd., Crawley, UK). Efficiency of individual siRNA knockdowns in T cells was evaluated by Western blotting. Primary T cells were transfected with the siRNAs also at 400 nM per reaction using Amaxa Human T cell Nucleofector kit (Lonza, Cologne, Germany).

Alternatively HSB2 T cells were transfected with full-length dominant negative human G protein alpha q, Q209L/D277N (Gαq, CloneID GNA0Q000X0), human G protein alpha 11, Q209L/D277N (Gα11, CloneID GNA11000X0), G-protein i2, G203T (Gαi2, CloneID GNAI12000T0) (UMR cDNA Resource Center, University of Missouri) (cDNA constructs, pCDNA3.1 vector control, all @10 µg cDNA per reaction. The strategy for the DN Gαq/11 cDNA constructs involved creation of xanthine nucleotide binding mutants of Gα11 and by analogy Gαq mutants that act in a dominant negative fashion by binding to their appropriate receptors and blocking GTP-mediated activation [33]. Additionally the Gαi2 DN mutant G203T has been described [34]. Flow cytometry following co-transfection of T cells with EGFP cDNA @ 0.1 µg cDNA per reaction revealed ∼50% T cells were successfully transfected.

Transfected cells were maintained in RPMI 1640 with 10% FCS for up to 48 h for siRNA-, and 24 h or 48 h, for cDNA-transfected HSB2 cells. Transfected T cells were routinely >90% T cell viable.

### LFA-1 Internalization Assay

This protocol was adapted from [35]. Glass coverslips (32 mm) were coated overnight with 3 µg/ml ICAM-1Fc, then blocked with 2.0% BSA. To biotinylate membrane proteins, washed T cells were re-suspended in 0.5 mg/ml EZ-link Sulpho-NHS-SS-Biotin (21331, Pierce, ThermoFisher Scientific, Loughborough, UK) at 25×10^6^ cells/ml and incubated on ice for 1 h. After washing, 4×10^6^ T cells in HBSS buffer were added to each ICAM-1-coated coverslip. Primaquine diphosphate (PQ) (160393, Sigma-Aldrich Ltd) at 300 µM was added and the cells incubated for 40 min at 37°C to allow internalization of receptors. To remove membrane bound biotin, fresh glutathione buffer (46 mM glutathione, 75 mM NaCl, 1 mM EDTA, 1% BSA, 75 mM NaOH) was added and the cells incubated on ice for 30 min. Controls for biotinylation of total LFA-1 were maintained in PBS.

To analyze biotinylated LFA-1, T cells were lysed with a standard buffer containing 0.2% NP40 buffer. Immunoprecipitation using anti-LFA-1 mAb 38 and subsequent blotting were performed as previously described [36]. Biotinylated LFA-1 was revealed by blot incubation with Streptavidin-HRP conjugate (RPN1231, GE Healthcare) in PBS/0.1% Tween 20 and ECL reagent (GE Healthcare). Samples were also probed with 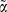
tubulin mAb and anti-mouse IgG-HRP Ab (GE Healthcare) to check for equivalent sample loading.

### Chemotaxis

For chemotaxis assays, T cells at 5×10^6^ cells/100 µl were allowed to migrate through 5 µm pore size Transwell insert wells coated with ICAM-1Fc as above (Corning, Acton, MA, USA). The lower wells contained either 600 µl RPMI 1640/0.1% BSA alone or medium plus 10 nM CXCL10 or CXCL12 (PeproTech EC Ltd, London, UK). After 90 min of incubation at 37°C and 5% CO_2_, inserts were discarded and the migrated T cells were counted by flow cytometry after recovery using ice-cold 5 mM EDTA/PBS. All samples were tested in triplicate.

### Video Microscopy

35 mm glass-bottom microwell dishes (MatTek Corp., Ashland, MA, US) or μ-slides VI (Ibidi GmbH, Martinsried, Germany) were coated overnight with 3 µg/ml ICAM-1Fc as above. The T cells (2×10^6^ cells/ml in HBSS with 20 mM HEPES (H.HBSS) were exposed to ICAM-1 for 10 min at 37°C and images captured using an Olympus MTV3 Inverted microscope using a 20× lens or Zeiss Axiovert 135TV Inverted microscope using a 63× lens plus AQM^2001^ Kinetic Acquisition Manager software (Kinetic Imaging Ltd). The cells were tracked at 15 sec intervals with Motion Analysis software (Kinetic Imaging Ltd, Bromborough, UK) and data analyzed using a Mathematica notebook (Wolfram Research, Long Hanborough, UK) developed by D. Zicha (Cancer Research UK).

Uropod attachment was quantified as previously reported [37]. Briefly individual live migrating T cells were observed using a visual assessment and scored by two observers. Analysis of the attachment status was accomplished by focus on both the T cell contact interface with ICAM-1 and the focal plane above this level.

### Confocal and TIRF Microscopy

13 mm round glass coverslips or glass bottomed MatTek dishes were pre-coated with ICAM-1-Fc as above. Washed T cells (2×10^5^ cells/sample) were added to coated coverslips for 30 min. Adherent cells were fixed with fresh 3% paraformaldehyde in Pipes buffer (pH8) for 5 min at RT, washed and fixed again in 3% paraformaldehyde in Borax, (pH 11, Sigma-Aldrich Ltd)[14]. Cells were then permeabilized with 0.1% Triton-X-100 for 5 min at 4°C. Autofluorescence was quenched using fresh sodium tetraborate (1 mg/ml, pH8) for 15 min at RT. Coverslips were incubated with primary mAbs overnight at 4°C, followed by Alexa488-goat anti-mouse IgG or Alexa546-goat anti-rabbit IgG (Invitrogen, Paisley, UK) for 45 min. Images were acquired on a Zeiss Laser Scanning Microscope LSM 710 or 780 using Zen software and x63 DIC oil lens.

The polarity of the T cells was determined by examining their morphology. A migrating T cell displays a spreading lamellipodium at the leading edge and an elevated uropod at the trailing edge, although HSB2 cells attach less ideally that T lymphoblasts. We have confirmed the assignments in previous studies of both leading edge (F-actin cross-linking; α-actinin localization) and trailing edge (increased α-tubulin and ICAM-1/3 distribution)[14], [36], [38].

TIRF images were acquired using a TIRF microscope system (Cell R, Olympus) based on an inverted microscope (IX 81, Olympus), TIRF illuminator with 488 nm and 561 nm lasers (Olympus), an objective (UAPO 150× TIRFM, NA 1.45, Olympus) and a sensitive EMCCD camera (iXon3 897, Andor) using Xcellence software (Cascadell, Photometrics).

For quantification of the fluorescence using the microscope software, an arbitrary mean fluorescence value per designated cell area was determined. This was done by applying a filter between 500–1000 mHz to exclude both the background and saturated signals, leaving the positive signal of interest and generating the mean fluorescence intensity (MFI) ± s.d.

The extent of co-localization of different markers was analyzed using Image J software and JACoB analysis. Co-localization was measured using the Manders coefficient to evaluate the overlap in fluorescence.

### Detection of Cell Membrane LFA-1 Using Laser Scanning Cytometry

Epitope expression and the area of spread contour of T cells mounted on ICAM-1 coverslips were calculated based on their fluorescence using a Laser Scanning Cytometer (CompuCyte, Mass, USA) and WinCyte version 3 software (Compucyte). The technique has the advantage of allowing investigation of membrane expression of epitopes on lymphocytes that are adhered to and spread on the LFA-1 ligand ICAM-1. The approach was to allow cell attachment and migration followed first by cell fixing and labelling with anti-LFA-1 mAb 38 conjugated to AlexaFluor-488, then by permeabilization with 0.1% Triton X100 (5 min on ice) and labeling with Phalloidin-PE. The threshold level for measurement was set using the signal from Phalloidin-PE so that individual cells could be segmented and assigned an area value (microns squared). Within this area, the total integrated AF488 signal was measured by summation of the value of AF488 fluorescence for each pixel (10-bit scale per pixel). In Fig. 7C, we measured the change in AF488 measurements per average cell area of T cells treated with cDNA constructs of DN Gαq/11 compared with control cDNA. 6000 T cells were measured per coverslip.

### Statistical Analysis

The migration and other assays are presented as mean±s.d. The unpaired Student’s *t* test was performed using GraphPad Prism software version 5 for Macintosh computers. The following significant differences are as indicated: *, *P*<0.05; **, *P*<0.01 and ***, *P*<0.001.

## Supporting Information

Figure S1 **Chemotaxis of HSB2 T cells to CXCL12.** Transwell assay showing the chemotactic response to CXCL12 of HSB2 T cells transfected with Gαq/11 or control cDNAs, n = 3 experiments.
(EPS)

Figure S2 **Ineffectiveness of Gαq/11 blockade on T cell adhesion in static and shear flow assays.** (**A**) HSB2 T cells treated with either control or Gαq siRNAs were tested at a shear force of 1 dyne using chambers coated with E-selectin alone to detect rolling cells and E-selectin and ICAM-1 together to detect both slow rolling and stable adhesion due to LFA-1 engagment; n = 3 experiments; (**B**) Static ICAM-1 adhesion assay of HSB2 T cells treated either with DN Gαq, DN Gα11 or a combination of both cDNAs; results are representative of n = 5 experiments.
(EPS)

Video S1 **HSB2 T cells migrating on ICAM-1 after transfection with control cDNA for 24 h.** T cells with trailing uropods are observed to be migrating. Each frame  = 1/10 sec representing 15 s real time.
(MOV)

Video S2 **HSB2 T cells migrating on ICAM-1 after transfection with DN Gαq/Gα11 cDNAs for 24 h.** Note the attached uropods on T cells attempting to migrate. Each frame  = 1/10 representing 15 s real time.
(MOV)
